# Ataxia-Telangiectasia Group D Complementing Gene (ATDC) Promotes Lung Cancer Cell Proliferation by Activating NF-κB Pathway

**DOI:** 10.1371/journal.pone.0063676

**Published:** 2013-06-12

**Authors:** Zhong-Ping Tang, Qian-Ze Dong, Quan-Zhe Cui, Paulie Papavassiliou, En-Di Wang, En-Hua Wang

**Affiliations:** 1 Department of Pathology, The First Affiliated Hospital and College of Basic Medical Sciences of China Medical University, Shenyang City, China; 2 Department of Pathology, Duke University Medical Center, Durham, North Carolina, United States of America; University Magna Græcia, Italy

## Abstract

Previous studies suggested Ataxia-telangiectasia group D complementing gene (ATDC) as an oncogene in many types of cancer. However, its expression and biological functions in non-small cell lung cancer (NSCLC) remain unclear. Herein, we investigated its expression pattern in 109 cases of human NSCLC samples by immunohistochemistry and found that ATDC was overexpressed in 62 of 109 NSCLC samples (56.88%). ATDC overexpression correlated with histological type (p<0.0001), tumor status (p = 0.0227) and histological differentiation (p = 0.0002). Next, we overexpressed ATDC in normal human bronchial epithelial cell line HBE and depleted its expression in NSCLC cell lines A549 and H1299. MTT and colony formation assay showed that ATDC overexpression promoted cell proliferation while its depletion inhibited cell growth. Furthermore, cell cycle analysis showed that ATDC overexpression decreased the percentage of cells in G1 phase and increased the percentage of cells in S phase, while ATDC siRNA treatment increased the G1 phase percentage and decreased the S phase percentage. Further study revealed that ATDC overexpression could up-regulate cyclin D1 and c-Myc expression in HBE cells while its depletion down-regulated cyclin D1 and c-Myc expression in A549 and H1299 cells. In addition, ATDC overexpression was also associated with an increased proliferation index, cyclin D1 and c-Myc expression in human NSCLC samples. Further experiments demonstrated that ATDC up-regulated cyclin D1 and c-Myc expression independent of wnt/β-catenin or p53 signaling pathway. Interestingly, ATDC overexpression increased NF-κB reporter luciferase activity and p-IκB protein level. Correspondingly, NF-κB inhibitor blocked the effect of ATDC on up-regulation of cyclin D1 and c-Myc. In conclusion, we demonstrated that ATDC could promote lung cancer proliferation through NF-κB induced up-regulation of cyclin D1 and c-Myc.

## Introduction

Lung cancer is one of the leading causes of all cancer-related deaths worldwide and, in particular, non-small-cell lung cancer (NSCLC) constitutes the majority of the diagnosed cases [1], [2]. Multiple factors including genetic, epigenetic and microenvironmental, play important roles in the survival and colonization of tumor cells at a distant tissue site, leading to the metastasis [3]. However, despite many experimental studies, an underlying molecular mechanism that governs the metastasis of individual tumors has not yet been fully understood. Due to the limited success of conventional therapies in achieving a long-term survival in lung cancer patients, research efforts have been focused on the biological pathways involved in tumor progression and neoplastic cell survival in order to identify potential therapeutic targets [4].

Ataxia-telangiectasia group D complementing gene (ATDC) is a member of the tripartite motif (TRIM) family [5]. TRIM proteins typically have a series of conserved domains including multiple zinc finger motifs and a leucine zipper motif. These proteins have been demonstrated to participate in cell growth regulation and development and have been implicated in several human diseases such as HIV infection and leukemia [6], [7]. In particular, TRIM proteins such as TRIM8, TRIM22, TRIM38 and TRIM40 have been reported to engage in regulating NF-κB activation [8]–[11]. ATDC, also known as TRIM29, was initially identified in a search for the gene responsible for the genetic disorder ataxia-telangiectasia and was found to possess radiosensitivity suppressor functions [12]. Subsequent studies showed that ATDC was overexpressed in multiple types of cancers including pancreatic, gastric, bladder, colorectal, ovarian and endometrial cancers, as well as in plasma cell myeloma [13]–[21]. Whereas, its expression was apparently reduced in several other tumors, such as melanoma, breast, prostate, head and neck cancers [22]–[27]. Only one report described increased ATDC mRNA expression in association with high histological grade, large tumor size, extent of tumor invasion and lymph node metastasis in gastric cancer [15]. However, to the best of our knowledge, the protein expression of ATDC and its relationship with clinicopathological factors in primary lung cancers have never been characterized.

A recent study in a pancreatic adenocarcinoma cell line demonstrated that ATDC interacts with Disheveled-2 and the components of β-catenin destruction complex to stabilize β-catenin and activate wnt signaling, a crucial pathway that promotes tumor progression in many types of cancer [13]. Other studies suggested that ATDC binds p53 in the cytoplasm to sequestrate it from nucleus resulting in down-regulation of its target gene p21 [28]. In the A431 human squamous carcinoma cell line, ATDC was noted to interact with the intermediate-filament protein vimentin and with an inhibitor of protein kinase C, thereby acting as a component of the protein kinase C signal transduction pathway [29]. Altogether, these previous studies suggest that ATDC may function as an oncogene to promote cancer cell proliferation and invasion. However, the biological roles of ATDC in lung cancer cells have not yet been determined.

In order to address the above questions, we checked ATDC expression and tissue distribution in non-small-cell lung cancer by immunohistochemistry and analyzed its association with clinicopathological parameters. We also investigated the roles of ATDC on cell proliferation and cell cycle progression using gain- or loss-of-function approaches. More importantly, we explored the potential mechanism by which ATDC functions to promote the proliferation of lung cancer cells.

## Methods

### Patients and Specimens

This study was conducted with the approval of the institutional review board at China Medical University. Written consent was given by the participants for their information to be stored in the hospital database for their specimens to be used in this study. And all clinical investigation has been conducted according to the principles expressed in the Declaration of Helsinki. The primary tumor specimens were collected from 109 patients with squamous cell carcinoma (SCC) or adenocarcinoma of the lung who underwent complete surgical resection in the First Affiliated Hospital of China Medical University between 2001 and 2004. Large cell carcinoma, adenosquamous cell carcinoma or other NSCLC subtypes were excluded from this study. Follow-up information was obtained by reviewing the patients’ medical records. None of the patients had received radiotherapy or chemotherapy before surgical resection, and all the patients were treated with routine chemotherapy after the resection. All 109 cases were reviewed for histological subtype, differentiation, and tumor stage. The histological diagnosis and grade were evaluated on haematoxylin and eosin-stained sections according to the World Health Organization (WHO) guidelines of classification. Of 109 cases, 46 (42.2%) were SCC and 63 (57.8%) were adenocarcinoma. Lymph node metastases were identified in 44 (40.4%) of the 109 patients. The p-TNM staging system of the International Union against Cancer (7th Edition) was used to classify the cases.

### Cell Culture and Treatment

NHBE, A549, H1299, H157 and H460 cell lines were obtained from American Type Culture Collection (Manassas, VA, USA). LH7 and LK2 cell lines were purchased from Shanghai Cell Bank of Chinese Academy of Science. The BE1 cell line was provided as a gift by Dr. J Zheng (Department of Pathology, Peking University School of Medicine). The BE1 cell line was established by Dr. J Zheng and Dr. WY Zhu in 1995 and now could be purchased from National Platform of Experimental Cell Resources for Sci-Tech (Peking, China). Cells were cultured in RPMI 1640 (Invitrogen, Carlsbad, CA, USA) containing 10% fetal calf serum. Cells were grown on sterile tissue culture dishes and were passaged every 2 days using 0.25% trypsin (Invitrogen). NF-κB inhibitor BAY 11-7082 (10 µM, 6 hours) was purchased from Sigma Aldrich.

### Immunohistochemistry

Surgically excised tumor specimens were fixed with 10% neutral formalin, embedded in paraffin and 4 µm thick sections were prepared. Immunostaining was performed using the avidin–biotin–peroxidase complex method (Ultra Sensitive TM, Maixin, Fuzhou, China). The sections were deparaffinized with xylene, rehydrated with graded alcohol series and boiled in 0.01 M citrate buffer (pH 6.0) for 2 minutes in an autoclave. Endogenous peroxidase activity was blocked using hydrogen peroxide (0.3%) before incubation of the sections with normal goat serum to reduce non-specific binding. Tissue sections were then incubated with ATDC rabbit polyclonal antibody (1∶150 dilution) (cat.17542-1-AP, Proteintech, IL, USA). Rabbit immunoglobulin (at the same concentration of the antigen specific antibody) (cat.NC-100, Maixin, Fuzhou, China) was used as a negative control. Immunohistochemical staining for Ki67 (1∶200 dilution) (cat.MAB-0129, Maixin, Fuzhou, China), c-Myc (1∶500 dilution) (cat.ab32, Abcam) and cyclin D1 (1∶100 dilution) (cat.2978, Cell signaling technology, Boston, MA, USA) were also performed following the manufacturers’ instructions. Staining for all primary antibodies was performed at room temperature for 2 hours. Biotinylated goat anti-mouse serum IgG or biotinylated goat anti-rabbit serum IgG (ready-to-use) (cat.KIT-9922, Maixin, Fuzhou, China) was used as the second antibody. After washing, the sections were incubated with horseradish peroxidase-conjugated streptavidin–biotin, followed by 3, 3′-diaminobenzidine tetrahydrochloride to develop peroxidase reaction. Counterstaining was done with hematoxylin, and the sections were then dehydrated with ethanol before being mounted.

Two investigators independently evaluated all tumor slides. Five random fields were examined per slide, and 100 cells were evaluated per high magnification field (400×). Normal bronchial epithelium in the tumor sections was used as an internal negative control. Immunostaining of ATDC was scored following a semi-quantitative scale by evaluating the staining intensity and percentage of cells in representative tumor areas as compared to that observed in the control cells. Positive immunostaining was defined as a distinct cytoplasmic staining of the tumor cells. The intensity of ATDC cytoplasmic staining was further stratified as 0 (no staining), 1 (weak) and 2 (strong). Percentage scores were designated as 1 (1–25%), 2- (26–50%), 3 (51–75%) and 4 (76–100%). The scores of each tumor sample were multiplied to give a final score of 0 to 8 and the total expression of ATDC was determined as either negative or low expression (-), if the total score was <4, and as positive expression or high expression (+), if the total score was ≥4.

The immunohistochemical staining for Ki-67 was evaluated and scored as percentage of the immunoreactive cancer cells, with a total of 500 tumor cells examined per slide. The median value of this series (35%) was used as a threshold value to stratify the tumors into the group with a low (<35%) index and the one with a high (≥35%) index of cell proliferation. The expression of c-Myc and cyclin D1 was defined as high level of expression or low level according to the previous criteria [30], [31].

### Quantitative Real-time PCR (SYBR Green Method)

Quantitative real-time PCR was performed using SYBR Green PCR master mix with a total volume of 20 µl in 7900HT Fast Real-Time PCR System (Applied Biosystems). The reaction condition is as follow: 95°C for 30 seconds, 40 cycles of 95°C for 5 seconds and 60°C for 30 seconds. A dissociation step was applied to generate a melting curve to confirm the specificity of the amplification. β-actin transcripts were amplified and served as the reference. The relative levels of gene expression were represented as ΔCt = Ct gene –Ct reference, and the fold change of gene expression was calculated by the 2-ΔΔCt method. Experiments were performed in triplicate.

The primer sequences were as follows:

β-actin forward, 5′-ATAGCACAGCCTGGATAGCAACGTAC-3′, β-actin reverse, 5′-CACCTTCTACAATGAGCTGCGTGTG-3′; ATDC forward, 5′-GCACCGGACACCATGAAGA-3′, ATDC reverse, 5′-GGAGACGAGGGCTGGTATGA-3′.

### Western Blot Analysis

Total proteins from NSCLC tissues and lung cancer cell lines were extracted in lysis buffer (Thermo Fisher Scientific, Rockford, IL) and quantified using the Bradford method. Fifty micrograms of protein were separated by SDS–PAGE (12%). After transferring, the polyvinylidene fluoride (PVDF) membranes (Millipore, Billerica, MA, USA) were incubated overnight at 4°C with the following antibodies: ATDC (1∶1000; cat.17542-1-AP, Proteintech), β-actin(1∶500; cat.612657), β-catenin (1∶1000; cat.610154) (BD Transduction Laboratories), cyclin A2 (1∶1000; cat.4656S), cyclin D1 (1∶1000; cat.2978), cyclin E1 (1∶800; cat.4129), CDK2 (1∶1000; cat.2546), CDK4 (1∶1000; cat.2906), CDK6 (1∶1000; cat.3136), p-Rb (Ser807/811) (1∶2000; cat.8516), P21 (1∶800; cat.2947), p-IκB (Ser32) (1∶500; cat.2859) (Cell signaling technology, Boston, MA, USA), c-Myc(1∶1000; cat.ab32, Abcam), active β-catenin (1∶500; cat.05-665, Millipore), p65 (1∶500; sc-8008, Santa Cruz). After incubation with peroxidase-coupled anti-mouse (cat.sc-2005) or rabbit (cat.sc-2004) IgG (Santa Cruz Biotechnology) at 37°C for 2 hours, bound proteins were visualized using ECL (Thermo Fisher Scientific) and quantified using BioImaging Systems (UVP Inc., Upland, CA, USA). The relative protein levels were calculated in reference to β-actin as the loading control.

### Plasmid Transfection and Small Interfering RNA Treatment

The pCMV6-ATDC plasmid was purchased from Origene. pCMV6 empty vector was used as a negative control. On-target plus siRNAs for ATDC (M-012409-01) and non-targeting siRNA #1 (D-001810-01) were purchased from Dharmacon (Lafayette, CO, USA). The plasmid and siRNA were transfected into cells using Attractene Transfection reagent (Qiagen, Hilden, Germany). Briefly, cells were seeded in a 6-well plate 24 hours before the experiment. The mRNA and protein levels of ATDC were determined 48 hours after transfection.

### Cell Proliferation Test and Colony Formation Assay

Cell proliferation assay was performed using Cell Counting Kit-8 solution (Dojindo, Gaithersburg, MD) according to the manufacturer’s protocol. Briefly, cells were seeded at a concentration of 5×10^3^ cells/100 µl/well in 96-well culture plates and treated with 10 µl/well of Cell Counting Kit-8 solution during the last 4 hours of the culture. Optical density of the wells was measured at 450 nm wave length using a microplate reader.

For colony formation assay, cells were planted into three 6-cm cell culture dishes (1×10^3^ cells per dish) and incubated for 12 days. Plates were washed with PBS and stained with Giemsa. The number of colonies with more than 50 cells was counted.

### Cell Cycle Analysis

Cells (5×10^5^) were seeded into a 6 cm tissue culture dish. After 12 hour incubation, cells were treated with serum starvation for 24 hours before transfection with indicated doses of plasmids or siRNA. At the indicated time points, cells were harvested, fixed in 1% paraformaldehyde, washed with phosphate buffered saline (PBS) and stained with 5 mg/ml propidium iodide in PBS supplemented with RNase A (Roche, Indianapolis, IN) for 30 minutes at room temperature. The stained cells were collected and analyzed using BD flow cytometry systems.

### Luciferase Reporter Assay

Reporter gene transfection and luciferase activity assay were performed as follow: cells in confluent growth on a 24 well plate were co-transfected with the firefly luciferase reporter (0.2 µg) along with the Renilla luciferase reporter (Promega Co) (0.02 µg) for 12 hours using an attractene reagent (Qiagen) according to the protocols provided by manufacturers. The reporter plasmids of TOP/FLASH, p53 and NF-κB were purchased from Biotime Biotechnology, China. The luciferase activity was measured in cellular extracts using a dual luciferase reported gene assay kit (Promega, CA, USA). The relative activity of the reporter gene was calculated by dividing the signals from firefly luciferase reporter by the signals obtained from Renilla luciferase reporter.

### Statistical Analysis

SPSS version 16.0 for Windows was used for all statistical analyses. The Chi-squared test was used to examine possible correlations between ATDC expression and clinicopathologic factors. The Student’s t-test was used to test the difference between the groups and p-values were based on the two-sided statistical analysis, with a p-value of <0.05 designating statistical significance.

## Results

### Overexpression of ATDC Protein in Non-small Cell Lung Cancer Tissues and its Relationship with Clinicopathological Factors

**Figure 1:**
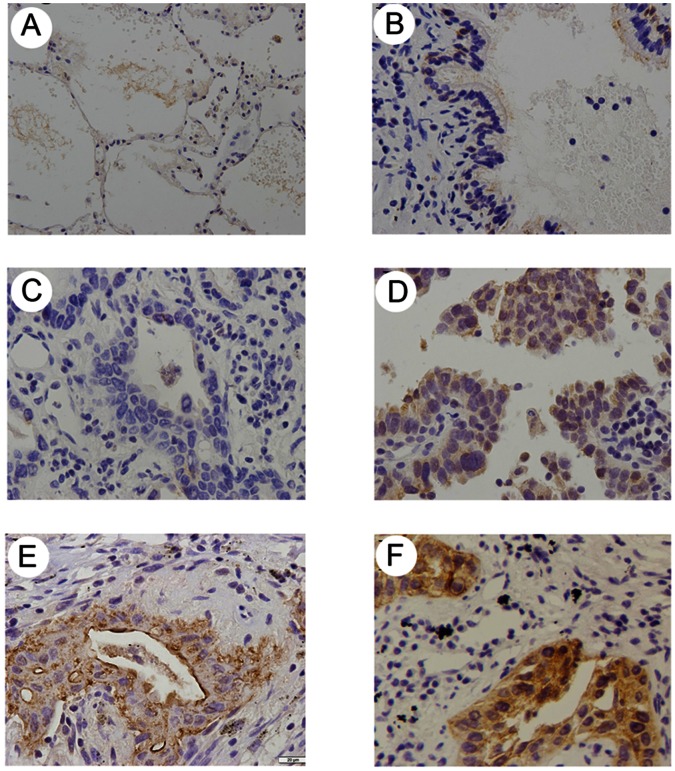
Immunohistochemical staining of ATDC in lung cancer tissue and paired normal lung tissue. A. Negative staining in normal pneumocytes in the alveoli of non-neoplastic lung tissue. B. Negative staining in normal bronchial epithelium in non-neoplastic lung tissue. C. Negative ATDC staining in lung adenocarcinoma. D. Weak ATDC staining in lung adenocarcinoma. E. Weak ATDC staining in lung squamous cell carcinoma. F. Strong ATDC staining in lung squamous cell carcinoma.

In order to investigate ATDC protein levels in lung cancer, we examined ATDC expression in a panel of 109 NSCLC specimens and 20 paired homologous normal lung tissues using immunohistochemistry. Overexpression of ATDC was noted in 62 (56.88%) out of 109 NSCLC specimens, and the ATDC protein was primarily localized in the cytoplasm of the tumor cells (Figure 1C–F). No or weak staining signals were detected in the normal lung tissues and the normal bronchial epithelia adjacent to the tumor (Figure 1A–B).

The relationship between ATDC expression and multiple clinicopathologic parameters was also analyzed. As shown in Table 1, a significant association was observed between ATDC overexpression and the extent of the primary tumor (T1 versus T2–T4∶42.11% versus 64.79%, p = 0.0227), histological differentiation (well differentiation versus moderate/poor differentiation: 31.43% versus 68.92%, p = 0.0002) and histological types (squamous cell carcinoma versus adenocarcinoma: 97.83% versus 26.98%, p<0.0001). No statistically significant difference was found between ATDC overexpression and patient age (p = 0.5449), gender (p = 0.3696), nodal status (p = 0.2327) or TNM stage (p = 0.5085).

**Table 1:** Expression of ATDC in the cases of NSCLC stratified by their clinicopathological parameters.

Characteristics	Number of patients	ATDC negative	ATDC positive	*P*
**Age**				
<60	59	27(45.76%)	32(54.24%)	0.5449
≥60	50	20(40.00%)	30(60.00%)	
**Gender**				
Male	61	24(39.34%)	37(60.66%)	0.3696
Female	48	23(47.92%)	25(52.08%)	
**Histology**				
Adenocarcinoma	63	46(73.02%)	17(26.98%)	<0.0001
Squamous cell carcinoma	46	1(2.17%)	45(97.83%)	
**Differentiation**				
Well	35	24(68.57%)	11(31.43%)	0.0002
Moderate- Poor	74	23 (31.08%)	51(68.92%)	
**TNM stage**				
I	48	19(39.58%)	29(60.42%)	0.5085
II+III	61	28(45.90%)	33(54.10%)	
**Tumor status**				
T1	38	22(57.89%)	16(42.11%)	0.0227
T2 T3 T4	71	25(35.21%)	46(64.79%)	
**Nodal status**				
N0	65	25(38.46%)	40(61.54%)	0.2327
N1 N2 N3	44	22(50.00%)	22(50.00%)	
**Ki67**				
Low	49	29(59.18%)	20(40.82%)	0.0022
High	60	18(30.00%)	42(70.00%)	
**cyclin D1**				
Low	39	25(64.10%)	14(35.90%)	0.0010
High	70	22(34.14%)	48(64.86%)	
**c-Myc**				
Low	72	37(51.39%)	35(48.61%)	0.0150
High	37	10(27.03%)	27(72.97%)	

### ATDC Promotes Cell Proliferation in Lung Cancer Cell Lines

**Figure 2:**
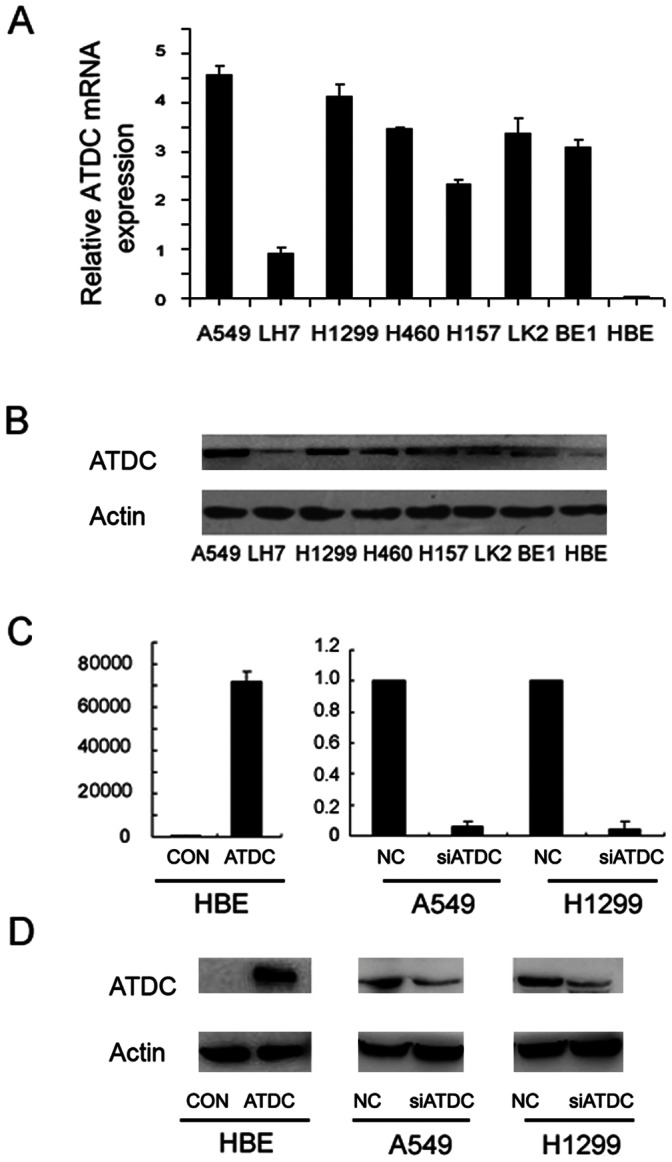
ATDC overexpression and knockdown in lung cancer cell lines. A. mRNA expression levels of ATDC analyzed by Real-time PCR in a panel of lung cancer cell lines. Note the highest expression is observed in A549 cell line and the lowest is seen in HBE cell. B. protein expression levels of ATDC analyzed by Western blot in a panel of lung cancer cell lines. Note a direct correlation of the protein level (B) with the transcripts (A) in each cell line. C. Real-time PCR analysis of ATDC overexpression efficiency in HBE cells and depletion efficiency in A549 and H1299 cells at a transcriptional level. D. Western blot analysis of ATDC overexpression efficiency in HBE cells and depletion efficiency in A549 and H1299 cells at a protein level. The band of lower MW in H1299 cells is a protein degradation product. Note a direct correlation of the protein level (D) with the transcripts (C) in each cell line.

Expression of ATDC was analyzed by real-time PCR and western blot assays in a panel of lung cancer cell lines and in a normal bronchial epithelial cell line HBE (Figure 2A–B). We found that both ATDC mRNA and protein expression levels in NSCLC cell lines were much higher than that in HBE, especially in A549 and H1299 cell lines. In order to investigate a possible role of ATDC in the cell growth of lung cancer, we transfected an ATDC cDNA expression construct into HBE cells and applied a pool consisting of three ATDC-targeting siRNAs to knockdown ATDC expression in both A549 and H1299 cells (Figure 2C–D). HBE cells demonstrated a significant increase in ATDC expression after ATDC transfection, while ATDC-specific siRNA efficiently blocked ATDC expression in A549 and H1299 cell lines.

**Figure 3:**
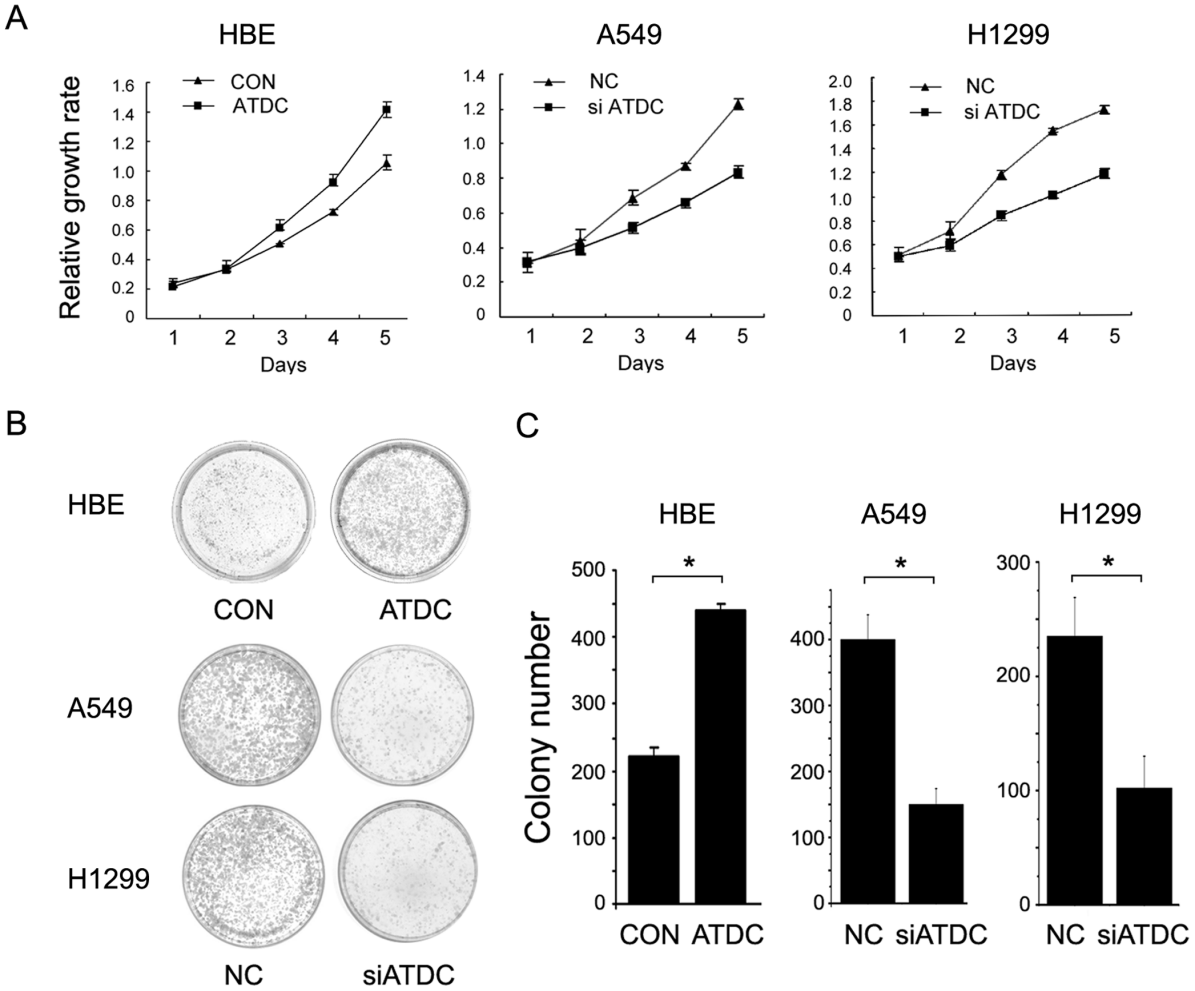
ATDC promotes cell proliferation in lung cancer cell lines. A. MTT assay shows that ATDC transfection in HBE cells promotes cell growth and ATDC knockdown in A549 and H1299 cells inhibits cell proliferation. B–C. Assessment of clonogenic potentials of the ATDC overexpressing cells and ATDC depleted cells by counting colony numbers. A remarkable increase in colony numbers was observed in the HBE cells with ATDC overexpression in comparison with control, and the number of colonies formed by A549 and H1299 cells treated with ATDC siRNA was far less than that of control cells. Columns in C represent the mean value of 3 duplicates; bars represent standard deviation [13]. * indicates a statistical significance in difference between the indicated 2 bar values (p<0.05).

MTT assay showed that transfection of ATDC cDNA into HBE cells promoted cell growth compared with control (transfection of empty vectors) (HBE control versus ATDC cDNA: 1.06±0.05 versus 1.41±0.05, p<0.05). The effect of ATDC on proliferation capacity was also analyzed by using colony formation assay. ATDC overexpression in HBE cells led to a remarkable increase in colony numbers (HBE control versus ATDC cDNA: 221±13 versus 441±9, p<0.05). On the other hand, the cell proliferation rate (A549 NC versus siATDC: 1.23±0.03 versus 0.84±0.03; H1299 NC vs siATDC: 1.72±0.01 versus 1.19±0.02; p<0.05) and colony formation ability (A549 NC versus siATDC: 400±12 versus 150±14; H1299 NC versus siATDC: 258±20 versus 119±17; p<0.05) were attenuated in A549 and H1299 cells with ATDC knockdown (Figure 3). These results altogether suggest that ATDC could modulate lung cancer cell proliferation.

### ATDC Facilitates G1/S Transition and Up-regulates Cyclin D1 and c-Myc Expression in Lung Cancer Cells

**Figure 4:**
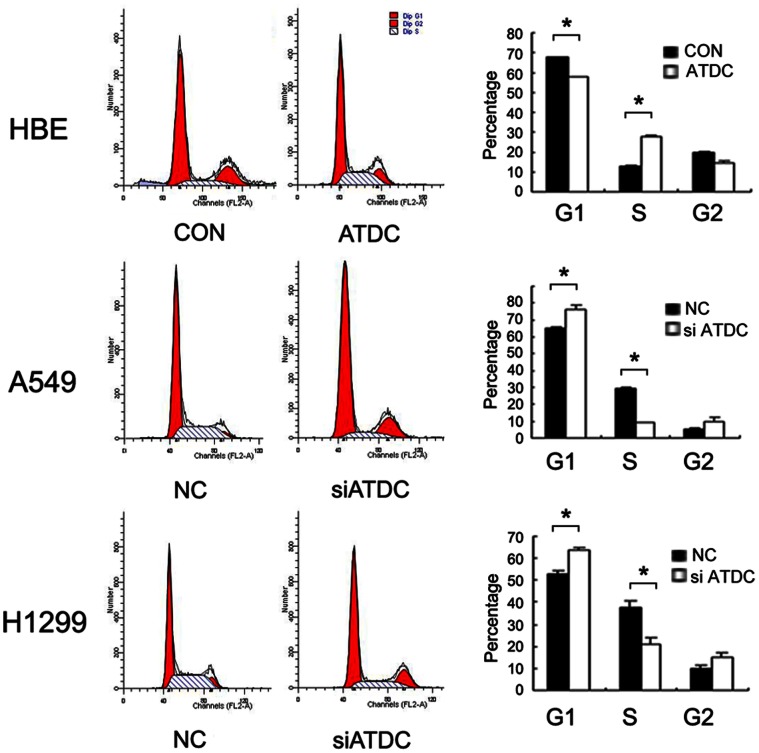
ATDC regulates cell cycle progression in lung cancer cells. Note ATDC overexpression in HBE cells increases S phase cells and decreases G1 phase cells (p<0.05 compared with control). ATDC knockdown in A549 and H1299 cells increases G1 phase cells and decreases S phase cells (p<0.05 compared with control).

Cell cycle analysis by fluorescence activated cell sorting (FACS) was performed in ATDC overexpressing HBE cells and in ATDC depleted A549 and H1299 cells (Figure 4). ATDC overexpression decreased the percentage of cells in the G1 phase (HBE control versus ATDC cDNA: 68.04%±0.04% versus 57.74%±0.51%, p<0.05) and increased the percentage of cells in S phase (HBE control versus ATDC cDNA: 12.48%±0.74% versus 27.62%±0.61%, p<0.05). Conversely, ATDC siRNA treatment increased the cells in the G1 phase (A549 NC versus siATDC: 64.99%±0.57% versus 75.99%±2.72%; H1299 NC versus siATDC: 52.66%±1.47% versus 63.80%±1.13%; p<0.05) and decreased the cells in the S phase (A549 NC versus siATDC: 29.59%±0.45% versus 9.24%±0.06%; H1299 NC versus siATDC: 37.55%±3.13% versus 20.80%±3.11%; p<0.05) compared with control. These results suggest that ATDC may promote cell cycle progression through the G1/S boundary.

**Figure 5:**
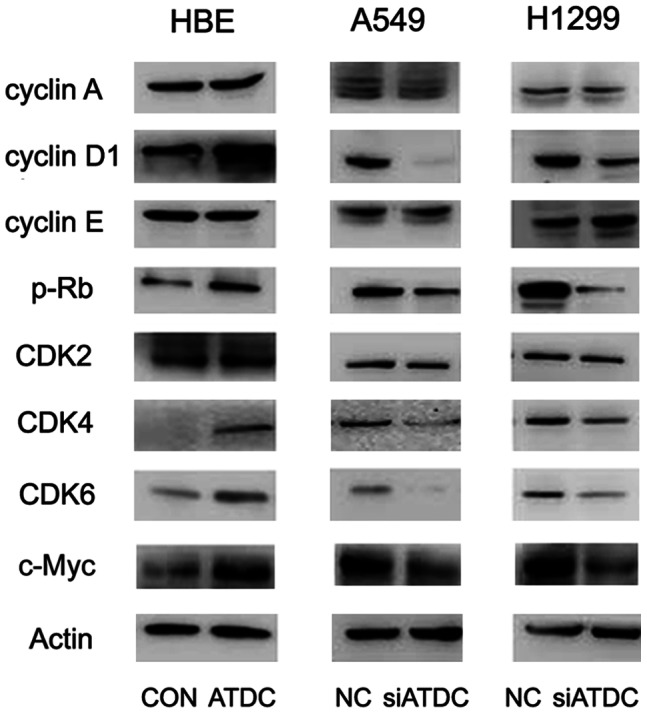
ATDC upregulates Cyclin D1 and c-Myc expression in lung cancer cells. Western blotting analysis reveals that ATDC transfection increases Cyclin D1 and c-Myc expression in HBE cells and knockdown of ATDC decreases the protein levels of cyclin D1 and c-Myc in both A549 and H1299 cells, without significant changes in Cyclin A and Cyclin E expression.

To investigate the mechanism of cell cycle progression regulated by ATDC in lung cancer, we examined the effect of ATDC on cyclin A, cyclin D1, cyclin E, CDK2, CDK4, CDK6, p-Rb and c-Myc in lung cancer cell lines. As shown in Figure 5, western blotting analysis revealed that ATDC overexpression up-regulated the protein levels of cyclin D1, CDK4, CDK6, p-Rb and c-Myc in HBE cells, while knockdown of ATDC decreased expression of these proteins in A549 and H1299 cells. Altogether, these results suggest that ATDC may promote G1/S transition via up-regulating cyclin D1 and c-Myc expression.

### ATDC Expression Correlates with Ki67 Labeling Index, Cyclin D1 and c-Myc Levels in NSCLC Tissues

**Figure 6:**
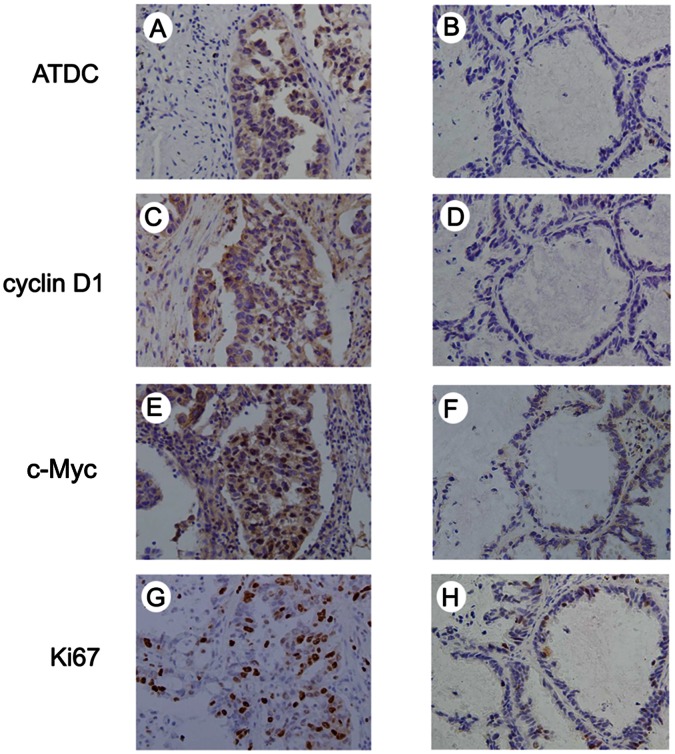
ATDC expression correlates directly with index of Ki-67 labeling and expression of cyclinD1 and c-Myc in NSCLC tissues. Immunohistochemical staining of ATDC (A and B), cyclin D1 (C and D), c-Myc (E and F) and Ki67 (G and H) in NSCLC specimens. A, C, E and G indicate a representative case with positive ATDC demonstrating a high level of cyclin D1 and c-Myc, a high labeling index of ki67, while B, D, F and H represent a case with negative ADTC and corresponding negative cyclin D1 and c-Myc staining or low level of ki67 labeling.

We examined the relationship between the level of total ATDC protein and the proliferation index (Ki67 labeling) in NSCLC. We found that cases with a high level of ATDC protein expression tended to show a high proliferation index, in comparison with those with a low level of ATDC (p = 0.0022) (Figure 6). Immunostaining for cyclin D1 and c-Myc was also performed in NSCLC specimens and their associations with ATDC expression were analyzed (Figure 6). As shown in Table 1, ATDC overexpression correlated with a high level of cyclin D1 (p = 0.0010) and c-Myc expression (p = 0.0150).

### ATDC Up-regulates Cyclin D1 and c-Myc in Lung Cancer Cells Independent of wnt/β-catenin or p53 Signaling Pathway

**Figure 7:**
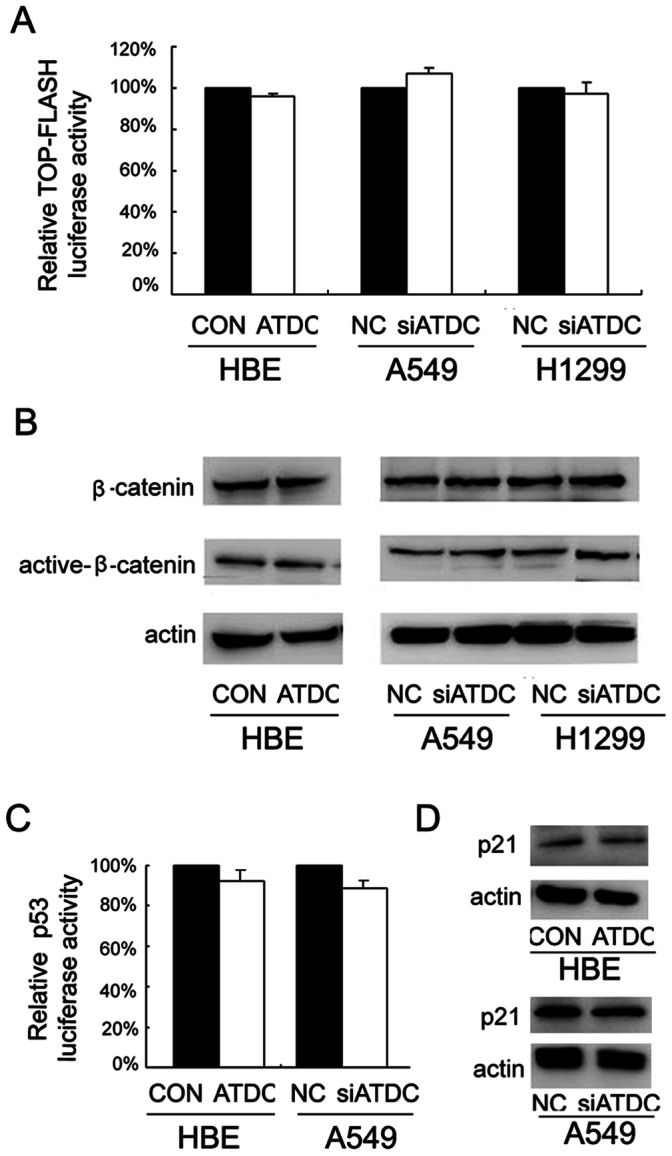
ATDC does not seem to be involved in regulating Wnt or p53 activity in lung cancer cell lines. A. There was no significant change of Topflash luciferase activity after ATDC transfection in HBE cells and after siRNA treatment in A549 and H1299 cells. B. There was no significant change of β-catenin and active β-catenin protein levels after ATDC transfection in HBE cells and after siRNA treatment in A549 and H1299 cells. C. ATDC transfection in HBE cells or its depletion in A549 cells did not change the level of p53 luciferase activity. D. ATDC transfection in HBE or its depletion in A549 cells did not change the protein level of p53 target gene p21.

Recent data shows that overexpression of ATDC leads to wnt signaling activation in pancreatic adenocarcinoma cell lines. This raises the question whether ATDC mediated up-regulation of cyclin D1 and c-Myc in lung cancer cells results from an activation of wnt signal transduction pathway in which cyclin D1 and c-Myc are crucial components. To test this, we measured total β-catenin and active β-catenin levels by western blot analysis, and examined the wnt activity by luciferase reporter assays in HBE cells overexpressing ATDC and in H1299 and A549 cells knocked-down of ATDC. No apparent changes of wnt activity and β-catenin levels were noted in these cells with altered levels of ATDC (Figure 7A–B). Therefore, the effect of ATDC on lung cancer cell proliferation seems to be independent of wnt/β-catenin signal transduction pathway.

Since ATDC was reported to increase cell proliferation via inhibition of p53 nuclear activities, we wondered if the ATDC mediated up-regulation of cyclin D1 and c-Myc in lung cancer cells could be due to its inhibitory effect on p53 protein. Among the 3 cell lines used above, H1299 cells have been well known to have p53 gene lost, while HBE and A549 cells express wild type of p53. Therefore, we evaluated the effect of ATDC on p53 activity in HBE and A549 cells. As shown in Figure 7C–D, overexpression of ATDC in HBE cells did not significantly change the activity of the p53-responsive luciferase reporter or the expression of p53 target gene p21. Neither change was noted in A549 cells depleted of endogenous ATDC. In conjunction with the fact that ATDC regulated cyclin D1 and c-Myc level in p53-null H1299 cells, we thought that the effect of ATDC on cell cycle progression in lung cancer cells might be independent of p53 activity.

### ATDC Up-regulates Cyclin D1 and c-Myc through Activation of NF-κB Pathway

**Figure 8:**
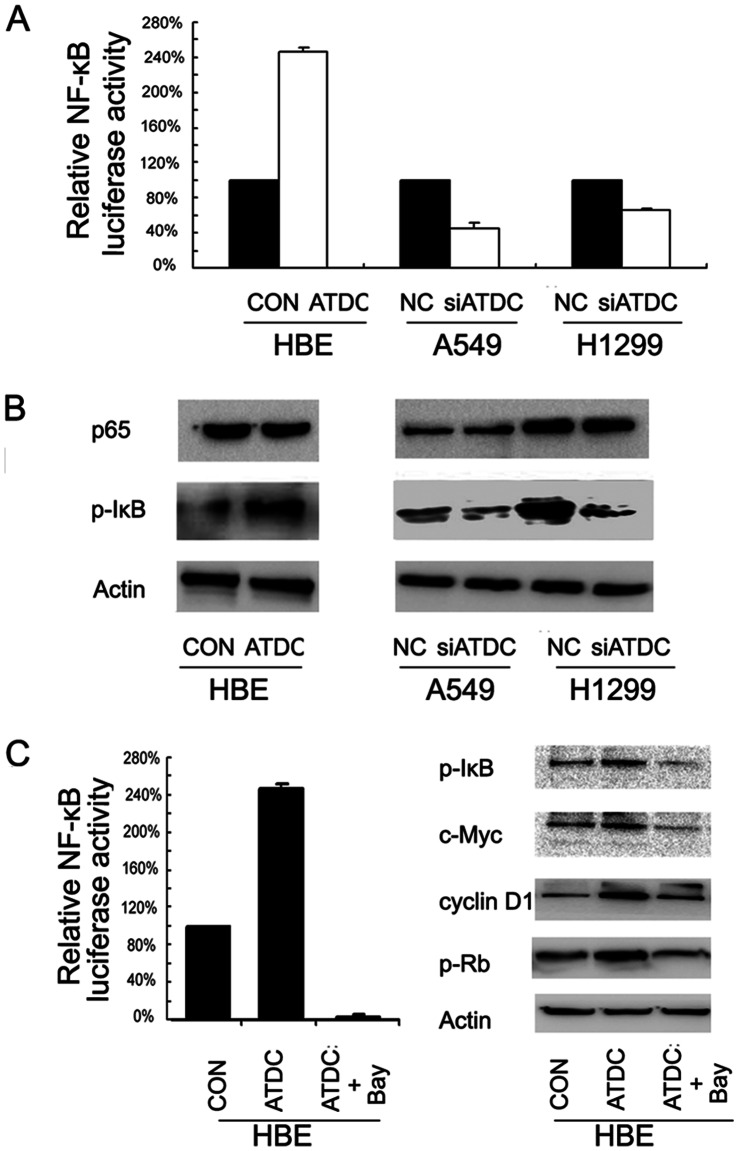
ATDC up-regulates Cyclin D1 and c-Myc via activation of NF-κB signaling pathway. A. ATDC overexpression up-regulated NF-κB reporter luciferase activity in HBE cells and ATDC depletion inhibited NF-κB reporter luciferase activity in both A549 and H1299 cells. B. ATDC transfection increased p-IκB expression in HBE cells and ATDC depletion decreased the level of p-IκB in A549 and H1299 cells. C. NF-κB inhibitor Bay 11-7082 completely blocked NF-κB reporter luciferase activity and reversed the effect of ATDC on cyclin D1, c-Myc and p-Rb up-regulation.

Since both cyclin D1 and c-Myc were downstream targets of NF-κB, we examined the protein expression of p65,p-IκB and NF-κB luciferase activity to assess if ATDC could regulate the activation of NF-κB pathway (Figure 8A–B). It appeared that ATDC overexpression in HBE cells up-regulated NF-κB reporter luciferase activity, and correspondingly, ATDC depletion in A549 and H1299 cells down-regulated its activity. On the other hand, the level of p-IκB was up-regulated in HBE cells transfected with ATDC and down-regulated in A549 and H1299 cells depleted of endogenous ATDC, although there was no significant change of total p65 level in these cells. These results suggest a possible involvement of NF-κB activation in ATDC induced up-regulation of cyclin D1 and c-Myc. Bay 11-7082, which inhibits phosphorylation and degradation of IkBa to block NF-κB activation, was also utilized in HBE cells transfected with ATDC to confirm the effect of NF-κB activation. As shown in Figure 8C, NF-κB inhibitor reversed the effect of ATDC on cyclin D1, c-Myc and p-Rb in HBE cells. Therefore, NF-κB activation may be a prerequisite for ATDC induced up-regulation of cyclin D1 and c-Myc.

## Discussion

ATDC, located at chromosome 11q22–23, belongs to the tripartite motif (TRIM) protein family (also known as the RBCC family) and was overexpressed in variety of different cancers [13]–[21]. Conversely, other studies reported a diminished expression of ATDC in a few other neoplasms, including breast, head and neck and prostate cancers [22]–[27]. Therefore, the expression pattern of ATDC seems to be paradoxically distinct among various types of cancers and the significance of ATDC in cancers, as well as its biological function in carcinogenesis and cancer cell progression, may vary depending upon the tissue origin of the neoplasm. A recent report described a correlation of ATDC mRNA expression with poor histological grade, large tumor size, extent of tumor invasion, and lymph node metastasis in gastric cancer [15]. In our study, we demonstrated that ATDC protein expression in lung cancer tissues was higher than that in normal lung tissues, and the expression of ATDC significantly correlated with histological type, tumor status and differentiation. These findings were generally consistent with previous studies which suggested a possible oncogenic function of ATDC in certain types of cancers.

Previous studies regarding the biological functions of ATDC exhibited that ATDC expression was associated with cell proliferation and tumor growth, suggesting that ATDC might act as a tumor promoter [13], [28]. To further investigate the functions of ATDC in lung cancer, we initially examined the expression level of ATDC in several lung cancer cell lines and immortalized human bronchial cell line NHBE by real-time PCR and western blot analysis. With these assays, we were able to confirm a minimal to essentially absent ATDC expression in HBE cells and a variable increase in ATDC expression in lung cancer cell lines at both transcriptional and translational levels. By applying gain- or loss-of-function approaches in HBE, A549 and H1299 cell lines in the current study, we hoped to shed light on the biological functions of ATDC in regulating neoplastic proliferation of lung cancer cells. Overexpression of exogenous ATDC promoted cell proliferation rate and colony formation in the HBE cell line that contained minimal endogenous ATDC. In addition, ATDC knockdown in the cells with high levels of ATDC showed impaired cell proliferation rate and colony formation. Cell cycle analysis showed that ATDC overexpression increased S phase cells and decreased G1 phase cells, suggesting that ATDC might facilitate G1 to S phase transition during cell cycle progression.

To better understand the role of ATDC in cell cycle regulation, we investigated the effect of ATDC overexpression and knockdown on a series of cell cycle-related molecules including cyclin A, cyclin D1, cyclin E, CDK2, CDK4, CDK6, p-Rb and c-Myc. Knockdown of ATDC reduced the protein levels of cyclin D1, p-Rb, CDK4, CDK6 and c-Myc in the 2 lung cancer cell lines with endogenous expression of ATDC. On the other hand, the expression of the above proteins was up-regulated after ATDC transfection in HBE cells. A few previous studies showed that cyclin D-Cdk4/6 complex partially phosphorylated Rb to inactivate its function as a transcriptional repressor and thus release transcription factor E2F, enabling the expression of target genes essential for G1 to S phase transition and DNA synthesis [32]. In accordance, overexpression of cyclin D1 shortened the G1 phase of the cell cycle and suppression of cyclin D1 prevented the cells from entry into S phase resulting in their arrest at G1 phase [33]–[35]. Furthermore, overexpression of c-Myc in growing cells was also reported to lead to a shortened G1 phase [36], whereas reduced expression causes lengthening of the cell cycle [37]. In our study, cell cycle arrest at G1 phase after ATDC knockdown in the 2 lung cancer cell lines (A549 and H1299) seems to be in keeping with corresponding down-regulation of cyclin D1 and c-Myc. Therefore, it may be that ATDC accelerates G1/S phase transition and promotes lung cancer cell proliferation by enhancing the expression of cyclin D1 and c-Myc.

In order to answer if the regulatory effect of ATDC on cell cycle progression in NSCLC is mediated by cyclin D1 and c-Myc upregulation in vivo, we examined Ki67 index, cyclin D1 and c-Myc expression in NSCLC tissues. Statistically significant correlations of ATDC expression were observed with level of proliferation index, cyclin D1 and c-Myc expression in neoplastic tissues. In conjunction with the data obtained from lung cancer cell lines, it is highly likely that ATDC may upregulate cyclin D1 and c-Myc and in turn accelerate cell cycle progression in NSCLC.

Cumulative evidence suggests that lung cancer cells use multiple, and perhaps redundant pathways to maintain their survival [38]. Recent investigations have revealed that ATDC modulates cancer progression through wnt/β-catenin activation or p53 inhibition [13], [28], [39], [40]. Cyclin D1 and c-Myc have been well documented to be regulated by nuclear β-catenin or a p53 target p21 [31], [41]–[43]. It is thus questioned if ATDC induced cyclin D1 and c-Myc up-regulation in lung cancer cells resultes from wnt activation or p53 inhibition. To test these possibilities, we evaluated the level of wnt/β-catenin and p53 activity in ATDC knockdown and overexpressing cells, and surprisingly, no significant changes of wnt/β-catenin and p53 activity were observed with alterations of ATDC level. Therefore, ATDC seems not being involved in regulating p53 or wnt signaling in lung cancer cell lines used in this study, despite its effect on cell proliferation and cell cycle progression.

NF-κB has been demonstrated to regulate many genes including cyclin D1 and c-Myc [44]–[46], and play important roles in tumorigenesis, cell proliferation and metastasis in cancers such as colon, lung and breast cancer [47]–[49]. TRIM proteins such as TRIM8, TRIM22 and TRIM38 have been reported to engage in NF-κB activation [8]–[10]. Interestingly, TRIM40 has been noted to physically bind to NEDD8 and promote the neddylation of IKKγ, which is a crucial negative regulator of NF-κB activation [11]. As a member of TRIM family, ATDC might possess a function in regulating NF-κB activity. As predicted, ATDC appeared to up-regulate p-IκB protein level in our further analysis. The activities of NF-κB luciferase reporter constructs were significantly enhanced by ATDC overexpression in HBE cells, and were diminished by ATDC knockdown in A549 and H1299 cells. This suggests a possible involvement of NF-κB pathway in ATDC induced cyclin D1 and c-Myc up-regulation and cell proliferation in lung cancer cells. When NF-κB inhibitor BAY 11-7082 was applied to HBE cells treated with ATDC transfection, the effects of ATDC on the levels of cyclin D1 and c-Myc were apparently abolished. These findings suggest that the role of ATDC in regulating cyclin D1 and c-Myc as well as cell cycle progression may be by means of NF-κB activation.

In conclusion, we, for the first time, demonstrate the expression and biological functions of ATDC in NSCLC. Briefly, ATDC could promote lung cancer proliferation through NF-κB induced up-regulation of cyclin D1 and c-Myc. The ATDC molecule may be used as a potential therapeutic target in the treatment of certain NSCLCs that express the protein. However, such a therapeutic approach needs to be further investigated and validated by additional clinical and experimental studies.
